# Clinical Applications of Cardiovascular CT: An Evolving Landscape

**DOI:** 10.3390/jcdd13050198

**Published:** 2026-05-06

**Authors:** David C. Rotzinger, Chiara Pozzessere, Guillaume Fahrni

**Affiliations:** Department of Diagnostic and Interventional Radiology, Cardiothoracic and Vascular Division, Lausanne University Hospital and University of Lausanne, 1011 Lausanne, Switzerland; chiara.pozzessere@chuv.ch (C.P.); guillaume.fahrni@chuv.ch (G.F.)

In its three decades of existence, cardiovascular computed tomography (CT) has profoundly transformed patient assessment and established itself as one of the most recommended non-invasive imaging modalities in contemporary cardiology and radiology practice [1]. From its early application in coronary artery calcium (CAC) quantification using electron beam CT in 1990 [2] to its current role as a multiparametric toolset integrating morphology, physiology, and tissue characterization, cardiovascular CT continues to reinvent itself at a remarkable pace, while refining patient safety [3]. The multiplicity of data retrievable from a single-modality examination is well illustrated in a recent meta-analysis that underscored the prognostic value of coronary CT angiography (CCTA); in a compelling assessment including fractional flow reserve CT (FFR-CT), high-risk plaque features, fat attenuation index, total plaque volume, low-attenuation plaque, and radiomics-derived parameters, the authors advocated CT for improved cardiovascular risk stratification [4]. This is before even considering artificial intelligence (AI), which, while still largely research-driven, is increasingly finding its way into clinical workflows and is well positioned to reshape cardiovascular CT practice in the years ahead [5]. While these advances reflect the significant research effort dedicated to detecting, characterizing, and managing coronary artery disease, cardiovascular CT applications extend far beyond coronary CCTA alone.

Beyond cardiac applications, CT angiography has established itself as a non-invasive tool for morphologic assessment of arterial vessels. Advances in imaging acquisition and reconstruction have enabled high-resolution 3D images for diagnosis and risk assessment of vascular diseases in acute and chronic settings, as well as for treatment planning, providing both detailed vascular mapping and measurements for device sizing in complex procedures [6]. The panel of vascular disorders that CT angiography can help diagnose and manage has been expanding continuously, currently encompassing degenerative, inflammatory, congenital, neoplastic, and traumatic pathology [7].

Reflecting this expansive scope, this Special Issue of the Journal of Cardiovascular Development and Disease, entitled “Clinical Applications of Cardiovascular Computed Tomography (CT),” brings together a diverse collection of original research articles, reviews, case reports, and study protocols that highlight the true breadth and depth of the field. A short summary of each contribution can be found in Table 1.

Naturally, the cornerstone of cardiovascular CT remains coronary artery disease (CAD) assessment. Camacho-Mondragon et al. provide a comprehensive focused review of three major clinical applications of cardiac CT for the practicing cardiac imager: CAC scoring, coronary CT angiography (CCTA), and preprocedural planning for structural heart interventions (Contribution 1). The authors summarize technical requirements and patient safety before systematically addressing the prognostic value of CAC scoring across diverse populations, the diagnostic and prognostic performance of CCTA, including high-risk plaque features and FFR-CT, and the indispensable role of CT in transcatheter valve procedures. Pushing the envelope further, the potential role of CCTA in acute coronary syndrome is discussed, an ongoing debate since the role of CT has evolved over the last few years and is increasingly integrated into major guidelines. Importantly, the review also covers emerging technologies, encompassing dual-energy imaging, photon-counting detector CT, and artificial intelligence, that are poised to further expand the clinical utility of the modality. This contribution serves as an accessible and timely reference for clinicians seeking to integrate cardiac CT into their daily practice.

Speaking of CAD prediction, preoperative cardiovascular risk stratification represents a clinically relevant yet underexplored application of CCTA and CAC scoring. Because of the scarcity of scientific data covering the specific setting of CAD in patients undergoing noncardiac surgery, no guidelines specifically recommend coronary imaging preoperatively. Kyriakoulis et al. present a narrative review examining the evidence supporting the use of CCTA and CAC score (CACS) in patients undergoing noncardiac surgery (Contribution 2). The authors demonstrate that both tools offer reliable perioperative and long-term major cardiovascular event (MACE) prediction, often matching or outperforming traditional imaging modalities such as dobutamine stress echocardiography and myocardial perfusion imaging. Notably, preoperative cardiac CT can help identify patients who carry an up to 12-fold risk of MACE during the 5 years following surgery. The review also highlights the emerging role of FFR-CT in this context and underscores the need for prospective comparative studies (CACS vs. CCTA and FFR-CT) to better define the optimal imaging strategy in the preoperative setting.

Beyond coronary artery disease, this Special Issue highlights the expanding role of CT in structural heart disease. Hammerer et al. investigated imaging predictors of the need for balloon post-dilatation (PD) following transcatheter aortic valve implantation (TAVI) in a retrospective single-center cohort of 585 patients (Contribution 3). PD can be required to manage incomplete TAVI expansion, where the valve frame fails to reach its intended diameter. Potential complications of underexpansion mainly include ischemic stroke, valve hypoattenuating leaflet thickening (HALT), and paravalvular regurgitation. Hammerer et al. identified aortic valve maximal systolic transvalvular flow velocity (echocardiography-derived) and aortic valve calcium density (cardiac CT-derived) as independent predictors of PD, while demonstrating that PD was not associated with increased procedural adverse events or impaired mid-term survival. These results reinforce the value of multimodal preprocedural CT- and echocardiography-based assessment in anticipating procedural complexity and optimizing patient outcomes in structural interventions.

The prognostic capabilities of cardiac CT extend well beyond coronary anatomy. Aoki et al. investigated the effectiveness of conventional CT-derived extracellular volume fraction (ECV) analysis for predicting MACE in patients with hypertrophic cardiomyopathy (HCM) (Contribution 4). ECV was derived from CT numbers [HU] measured in the myocardium (ΔHUm) and blood (ΔHUb) as well as the hematocrit (Hct) according to the following formula: ECV = (ΔHUm/ΔHUb)/(1 − Hct). In this multicenter retrospective cohort of 101 patients, left ventricular ECV on CT emerged as an independent predictor of MACE (*p* < 0.001), with an optimal threshold of 37.6% ECV yielding a sensitivity of 73% and specificity of 78%. This study is particularly noteworthy as it demonstrates that cardiac CT can provide tissue-level characterization, which is useful when MRI is limited by device incompatibility, arrhythmia, or claustrophobia. The ability to perform ECV analysis as an adjunct to standard coronary CT protocols, without additional contrast administration or extra ionizing radiation exposure, represents a meaningful advance in the multiparametric assessment of cardiomyopathies.

Epicardial adipose tissue (EAT) has gained increasing attention as a metabolically active compartment, with direct paracrine effects and bidirectional biochemical communication with the coronary vasculature and myocardium. However, full three-dimensional segmentation of epicardial fat is impractical, time-consuming, and incompatible with clinical workflows when performed manually. Lorusso et al. explored the feasibility of region-of-interest (ROI)-based epicardial fat density (EFD) measurement as a simplified alternative to full segmentation in 171 patients undergoing coronary CT (Contribution 5). Their results revealed significant regional variability in EFD across the aortic bulb, right posterolateral wall, and cardiac apex, with statistically significant differences compared to global EFD values they obtained using established semi-automatic segmentation software. While the study concludes that ROI-based measurements do not reliably reflect global EFD characteristics, it provides important methodological insights and highlights the need for dedicated segmentation tools to standardize EFD assessment in clinical practice. Future research integrating EFD with pericoronary fat attenuation index and plaque characterization may further enhance cardiovascular risk stratification.

The diagnostic versatility of CT is further illustrated by two contributions addressing less conventional applications. Tanaka and Yoshioka evaluate the performance of subtraction CT angiography (CTA) with volume position matching in 32 patients with lower extremity artery disease (LEAD) and severe arterial calcification (Contribution 6). When heavy calcification of the lower limb arteries is present, diameter stenosis assessment is typically challenging in the more distal vessels due to blooming artifacts. Tanaka and Yoshioka showed that compared to conventional CTA, subtraction CTA could overcome this limitation, dramatically reducing the proportion of uninterpretable arterial segments (2.0% vs. 25.2%) and achieving substantially higher diagnostic accuracy (0.885 vs. 0.657) against digital subtraction angiography (DSA) as the reference standard. These findings are particularly relevant for elderly patients and those with diabetes or end-stage renal disease, in whom Mönckeberg-type medial calcification frequently renders conventional CTA non-diagnostic. Subtraction CTA thus emerges as a practical and clinically valuable tool in this challenging population.

Capisizu et al. report a pilot study on the prevalence and characterization of coronary artery anomalies (CAAs) detected by CCTA in a Romanian cohort of 184 patients with chest pain (Contribution 7). A prevalence of 8.7% was observed, consistent with contemporary CCTA-based series, with anomalies of origin and course accounting for 3.8% and intramuscular bridging of the left anterior descending artery for 4.9%. The most frequent symptoms associated with CAA were chest pain in 52.7% and shortness of breath in 36.8% of patients. The presence of cardiac symptoms was statistically significantly associated with CAA (*p* = 0.045). This study is notable as the first to characterize CAA prevalence by CCTA in a southeastern Romanian population, contributing to the growing body of evidence supporting CCTA as the gold standard for CAA diagnosis and anatomical characterization.

The application of CT imaging to cerebrovascular disease is represented by the work of Arrarte Terreros et al., who investigated thrombus characteristics on acute stroke CT as predictors of early recanalization in transferred patients with anterior circulation large vessel occlusion (LVO) (Contribution 8). Using baseline non-contrast CT and CT angiography data from 81 early-recanalized patients compared with 322 persistent LVO patients from the MR CLEAN Registry, the authors demonstrate that intravenous thrombolysis administration, thrombus distality, and lower thrombus density were independent predictors of early recanalization, with a model AUC of 0.77. These findings highlight the potential of CT-based thrombus characterization to optimize repeated-imaging workflows and reduce ineffective inter-hospital transfers, with direct implications for resource allocation and patient safety in acute stroke management.

A rare but instructive case of aorto-esophageal fistula (AEF) caused by a vascular malformation is reported by Zhang et al. (Contribution 9). The case illustrates the critical diagnostic role of CT angiography in identifying the bleeding source, characterizing the vascular anomaly, and guiding emergency surgical planning in a condition with extremely high mortality. The authors emphasized that CTA’s non-invasive nature and high spatial resolution make it the preferred diagnostic modality when AEF is clinically suspected, avoiding the risk of precipitating fatal hemorrhage during endoscopy. This case expands the available literature covering the spectrum of primary AEF and reinforces the importance of attempting to map the vascular feeders in patients presenting with unexplained massive gastrointestinal bleeding prior to intervention.

Finally, this Special Issue includes a prospective randomized controlled trial protocol addressing a fundamental technical challenge in CCTA: motion artifacts. Fahrni et al. present the FAST-CCT trial, which aims to evaluate whether ultra-fast gantry rotation (0.23 s/rot) reduces coronary motion artifacts and improves diagnostic image quality compared to standard rotation speed (0.28 s/rot) in patients undergoing aortic stenosis work-up without β-blocker premedication (Contribution 10). This is a particularly relevant population, as β-blockers are frequently contraindicated in aortic stenosis, and motion artifacts remain a substantial limitation of CCTA in this setting. The trial’s prospective, randomized design and rigorous power analysis position it to generate high-quality evidence with direct implications for clinical practice and scanner protocol optimization.

Taken together, the contributions assembled in this Special Issue reflect the remarkable versatility of cardiovascular CT across a wide spectrum of clinical scenarios, from primary prevention and preoperative risk stratification to structural heart disease, cardiomyopathy, peripheral vascular disease, cerebrovascular emergencies, and rare vascular malformations. They also underscore the ongoing technological evolution of the modality, from subtraction algorithms and ECV mapping to ultra-fast gantry rotation and AI-assisted image analysis. As cardiovascular CT continues to expand its diagnostic and prognostic reach, the challenge ahead lies in translating these advances into accessible, cost-effective, and radiation-conscious clinical practice. We hope that this collection will serve as a valuable resource for clinicians, researchers, and imaging specialists and will stimulate further investigation into the many open questions that remain in this dynamic field.

We are grateful to all authors for their outstanding contributions, to the reviewers for their rigorous and constructive evaluations, and to the editorial team of the Journal of Cardiovascular Development and Disease for their support throughout this process.

## Figures and Tables

**Table 1:** Summary of the ten contributions included in this Special Issue.

Authors	Title	Type	Sample	Main Findings/Conclusions
Camacho-Mondragon et al. (Contribution 1)	Clinical Applications of Cardiac Computed Tomography: A Focused Review for the Clinical Cardiologists	Review	N/A	Cardiac CT has evolved into a comprehensive multiparametric modality integrating anatomy, function, and prognosis; innovations (photon counting, AI, dual energy) are expanding its diagnostic and prognostic role.
Kyriakoulis et al. (Contribution 2)	Coronary Computed Angiography and Coronary Artery Calcium Score for Preoperative Cardiovascular Risk Stratification in Patients Undergoing Noncardiac Surgery	Narrative review	N/A	CCTA and CACS (alone or combined) reliably predict perioperative and long-term MACE, often matching or surpassing traditional stress imaging; integration with a revised cardiac risk index further improves risk stratification; routine use is not yet recommended, but selective use is supported by guidelines.
Hammerer et al. (Contribution 3)	Importance of Imaging Assessment Criteria in Predicting the Need for Post-Dilatation in Transcatheter Aortic Valve Implantation with a Self-Expanding Bioprosthesis	Retrospective single-center	585 TAVI patients	Peak aortic valve velocity (AV Vmax) and aortic valve calcium density—not total aortic valve calcium score—independently predicted post-dilatation. Post-dilatation did not increase short/mid-term adverse events or mortality; it can be performed safely when indicated.
Aoki et al. (Contribution 4)	Extracellular Volume Fraction Analysis on Cardiac Computed Tomography Is Useful for Predicting the Prognosis of Hypertrophic Cardiomyopathy	Retrospective multicenter cohort	101 hypertrophic cardiomyopathy patients	LV-ECV and LVEF were independent MACE predictors; LV-ECV ≥ 37.6% identified significantly higher-risk patients (AUC 0.79). CT-derived ECV is a useful quantitative risk tool, especially when MRI is contraindicated.
Lorusso et al. (Contribution 5)	Evaluating Epicardial Fat Density Using ROI-Based Analysis: A Feasibility Study	Retrospective feasibility study	171 patients undergoing CCTA	Significant regional variability in epicardial fat density (aortic bulb, apex, posterolateral wall) vs. global was found; ROI-based measurements do not reliably reflect global epicardial fat density; dedicated segmentation software remains necessary.
Tanaka et al. (Contribution 6)	Subtraction CT Angiography for the Evaluation of Lower Extremity Artery Disease with Severe Arterial Calcification	Prospective diagnostic accuracy study	32 lower extremity artery disease patients (640 segments)	Subtraction CTA markedly reduced uninterpretable segments (2.0% vs. 25.2%) and achieved higher accuracy (0.885 vs. 0.657); this is particularly valuable in critical limb ischemia with heavy calcification.
Capisizu et al. (Contribution 7)	A Pilot Study on the Role of Computed Tomography in the Management of Patients with Coronary Artery Anomalies in Romania	Retrospective pilot study	184 patients undergoing CCTA	Coronary artery anomaly prevalence was 8.7% (3.8% origin/course; 4.9% intramuscular bridging of LAD). Significant association with cardiac symptoms was present; there was no association with calcium score. This supports wider CCTA use for coronary artery anomaly detection.
Arrarte Terreros et al. (Contribution 8)	Thrombus Imaging Characteristics to Predict Early Recanalization in Anterior Circulation Large Vessel Occlusion Stroke	Retrospective cohort and prediction model	81 early recanalization and 322 persistent large vessel occlusion stroke patients (MR CLEAN Registry)	Intravenous thrombolysis administration, greater distance to thrombus (distal occlusion), and lower thrombus density predicted early recanalization (AUC 0.77). Thrombus imaging may optimize repeated-imaging workflow in transferred stroke patients.
Zhang et al. (Contribution 9)	Aorto-Esophageal Fistula Caused by Vascular Malformation: A Case Description and an Analysis of the Literature	Case report and literature review	1 patient (60-year-old male)	CTA precisely identified the anomalous vessel and fistula, guiding emergency endovascular repair. This highlights the essential role of CTA in diagnosing aorto-esophageal fistula and the need for early surgical intervention, given the high mortality.
Fahrni et al. (Contribution 10)	Investigating the Influence of High-Speed Gantry Rotation in Cardiac CT on Motion Artifacts in Aortic Stenosis Patients Not Premedicated with β-Blockers: The FAST-CCT Randomized Trial Protocol	Prospective single-center RCT protocol	Planned 142 patients ≥ 50 years (aortic stenosis work-up, no β-blockers used for CT)	Study protocol aimed to determine whether faster gantry rotation eliminates the need for β-blocker premedication and improves CCTA image quality, diagnostic accuracy vs. invasive coronary angiography, and radiation dose. Results pending.

Acronyms; AI = artificial intelligence; AUC = area under the receiver operating characteristic curve; CACS = coronary artery calcium score; CCTA = coronary computed tomography angiography; CTA = computed tomography angiography; DSA = digital subtraction angiography; ECV = extracellular volume; LAD = left anterior descending; LV-ECV = left ventricular extracellular volume fraction; LVEF = left ventricular ejection fraction; MACE = major adverse cardiovascular events; MR CLEAN = Multicenter Randomized Clinical Trial of Endovascular Treatment for Acute Ischemic Stroke in the Netherlands; RCT = randomized controlled trial; ROI = region of interest; TAVI = transcatheter aortic valve implantation.
